# Radiotherapy plus immune checkpoint inhibitor in prostate cancer

**DOI:** 10.3389/fonc.2023.1210673

**Published:** 2023-07-21

**Authors:** Tianjie Li, Xinye Qian, Jinyang Liu, Feng Xue, Jing Luo, Guanqun Yao, Jun Yan, Xiaodong Liu, Bo Xiao, Jianxing Li

**Affiliations:** ^1^ School of Clinical Medicine, Tsinghua University, Beijing, China; ^2^ Department of Urology, Beijing Tsinghua Changung Hospital, Beijing, China; ^3^ School of Medical, Tsinghua University, Beijing, China; ^4^ Department of Urology, The First Affiliated Hospital of Kunming Medical University, Kunming, China

**Keywords:** radiotherapy, immune checkpoint inhibitor, prostate cancer, cold tumor, toxicity

## Abstract

The immune checkpoint inhibitor (ICI) is a promising strategy for treating cancer. However, the efficiency of ICI monotherapy is limited, which could be mainly attributed to the tumor microenvironment of the “cold” tumor. Prostate cancer, a type of “cold” cancer, is the most common cancer affecting men’s health. Radiotherapy is regarded as one of the most effective prostate cancer treatments. In the era of immune therapy, the enhanced antigen presentation and immune cell infiltration caused by radiotherapy might boost the therapeutic efficacy of ICI. Here, the rationale of radiotherapy combined with ICI was reviewed. Also, the scheme of radiotherapy combined with immune checkpoint blockades was suggested as a potential option to improve the outcome of patients with prostate cancer.

## Introduction

The immune checkpoint inhibitor (ICI) is believed to be promising for tumor patients (1). It could restore the antitumor function of cytotoxic T cells by blocking immune checkpoints, like PD-1 and CTLA-4 (2). However, ICI monotherapy is approved and useful for some cancers (e.g., breast, lung, kidney, bladder) (3) but has a very limited role in other cancers, with an ORR rate of around 3%–5% in prostate cancer (4), around 10%–13% in pancreatic cancer (5, 6), etc. Several clinical trials have explored the use of single-agent immune checkpoint inhibitors for the treatment of metastatic castration-resistant prostate cancer (mCRPC). The benefit seems to be restricted to a subset of patients (7). In a retrospective series of 11 patients with mCRPC characterized by MSI-H/dMMR who were treated with anti–PD-1/PD-L1 inhibitors alone or in combination with another CPI, the ORR was 50%; 54.5% of the patients demonstrated a PSA drop of higher than 50%, and four of these patients had a PSA decline of more than 99% (8). In 2017, the FDA approved pembrolizumab for the treatment of unresectable and metastatic tumors with an MSI-high (MSI-H) status or dMMR after progression on standard lines of treatment (9). Low incidence of TMB is one of the most important factors for ICI therapy failure (10). Behind this perspective, the true mechanism of ICI therapy is based on the antitumor function of CD8+ cytotoxic T lymphocytes. To fight against tumor, the human immune system must first recognize tumor cells with neo-antigens; then, T cells need to move to the tumor sight and contact with tumor cells to provide their antitumor effect. However, in the meantime, tumors would develop multiple mechanisms (mainly including T-cell priming dysfunction and T-cell homing dysfunction) to escape the hunt from tumor-reactive T cells (11). These tumors are called “Cold” tumors, tumors with a specific tumor microenvironment which causes the failure of ICI therapy (12).

**Figure 1:**
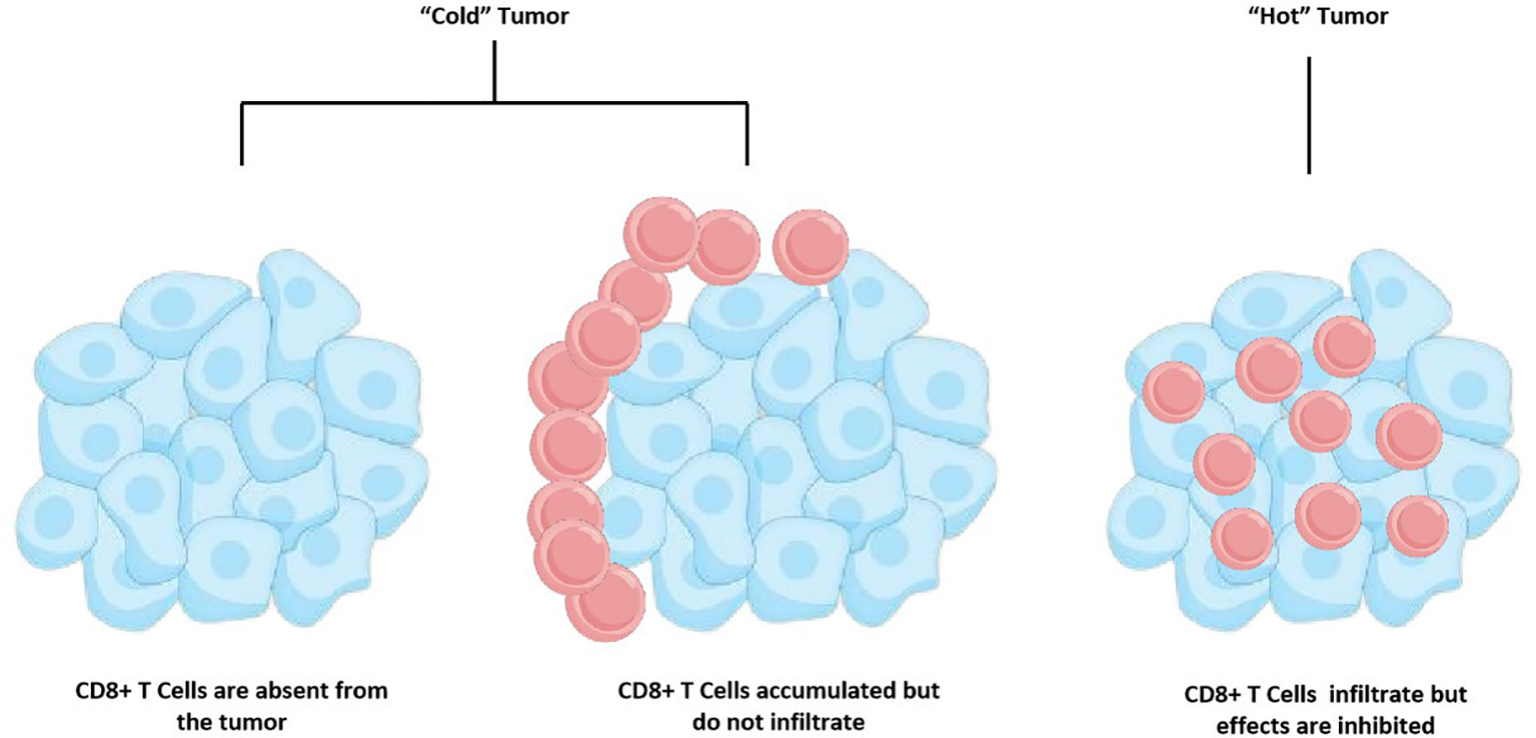
Tumor immune phenotypes.

“Cold” tumors have a limited infiltration of cytotoxic T lymphocytes (CTLs), a high activity of suppressor immune cells, and a poor response to ICI therapy. “Hot” tumors, on the opposite, are tumors that respond better to ICI therapy due to high infiltration of T cells and low activity of suppressor immune cells (**Figure 1**). “Cold” tumors are also called infiltrated excluded, non-inflamed, or non-immune reactive tumors (13). It is considered that prostate cancer is one of the “Cold” tumors (14).

Prostate cancer is the second most common cancers among men, after melanoma (15, 16). After definitive therapy, high-risk prostate tumors may have a recurrence and metastatic rate of around 30% (17). Although androgen deprivation therapy (ADT) is a standard therapy for prostate cancer in advanced stages, the tumor would still progress eventually. Around 70%–80% of prostate cancer would metastasize into the bone, which worsens the prognosis for prostate cancer patients (16). The immunologic landscapes of prostate cancer allow limited infiltration of immune cells into the tumor microenvironment (TME). The dysfunction of the immune system prevents it from initiating an effective response against tumors (18). Consequently, prostate cancer has a poor rate of response to ICI therapy (19).

Radiotherapy increases the levels of immune checkpoints (PD-1, CTLA-4) locally in the tumor by enhancing the activation and infiltration of immune cells into the TME and the level of PD-L1 expression on the surface of tumor cells (20). Moreover, immune check inhibitors can restore the antitumor activity of T cells by binding PD-1, CTLA, and PD-L1 and lower the immune escape of tumor cells (21). Consequently, radiotherapy and immune checkpoint inhibitor therapy are mechanistically complementary, and their combination may provide favorable outcomes in “Cold” tumor treatments. To improve the therapeutic effect of immune checkpoint blockade in prostate cancer, in this paper, radiotherapy is suggested as a preferable option for combined immune checkpoint inhibitor therapy to achieve a better therapeutic effect in patients with prostate cancer.

## Mechanisms of the “cold” tumor resisting ICI

The primary characteristic of a “cold” tumor is the absence of T-cell infiltration, which leads to low response rates of ICI therapy. Either defective T-cell priming or defective T-cell homing to the tumor bed is the reason for the failure of T-cell infiltration.

## T-cell priming dysfunction

Lack of tumor antigens and defects in tumor antigen presentation machinery are two main reasons for defective T-cell priming.

Lack of tumor antigens is the most direct cause of T-cell priming disorders. There are two categories of targeted tumor antigens: non-mutated self-antigens and neoantigens (22). Self-antigens, such as tumor-associated antigens (TAAs) and cancer/testis antigens (CTAs), are overexpressed non-mutated proteins in tumor cells. Neoantigens, also called tumor-specific antigens (TSAs), are specific mutated proteins in tumor cells. The recognition of tumor neoantigens by the immune system might promote T-cell priming and infiltration to the tumor region, which may result in antitumor responses (23).

This leads to tumor mutation burden (TMB), the total amount of non-synonymous single-nucleotide mutations in a tumor, a potential predictor for the response of ICI therapy. It is generally believed that tumors with a higher TMB would carry a higher neoantigen load that could be recognized by the immune system, making greater T-cell infiltration (24). Multiomics network analysis has further proved that higher mutation or neoantigen burden was positively correlated with higher CTL infiltration in various solid tumors, whereas lower mutational or neoantigen burden might be correlated with the lack of CTL infiltration and the failure of ICI therapy (25). However, kidney cancer presents with low TMB incidence, which showed 40% of response rate to ICI therapy (26). In gastric cancer, no difference in response rate was observed between TMB-H (16.7%) and TMB-L (16.2%) tumors treated with ICI (27). This evidence suggests that there are many other factors determining ICI response or resistance.

Damage to the machinery responsible for processing and presenting tumor antigens is another important cause of T-cell priming or homing defects in the tumor bed (APM). Normally, antigen-presenting cells (APCs) could express the tumor neoantigen peptide–MHC class I complex on its surface after recognition. However, impairs in the APM pathway, like downregulation of MHC-I molecule expression, could lead to the failure in presenting antigen peptide-MHC class I complexes even though tumor-specific antigens were present. During antigen processing and presentation, transporters associated with antigen processing (TAP) transport cytosolic cleaved antigens to the endoplasmic reticulum to bind with MHC molecules. Therefore, the deletion of TAP could also cause antigen presentation process failure, which further affects the priming of T cells (28). Beta-2-microglobulin (B2M), the invariant chain of the MHC, is critical for the successful folding and transport of MHC-I to the cell surface (29). Knocking down the B2M gene in human carcinoma cell lines led to the absence of MHC-I molecules expressed on their surface and the failure of tumor-specific T-cell recognition and cytotoxicity (30). These findings suggest that defects in the APM pathway would interfere with T-cell priming and the effectiveness of ICI therapy despite the presence of tumor antigens.

Dysfunction of dendritic cells (DCs) could also impair T-cell priming. DC activation requires pattern recognition receptors (PRRs), which are found on the surface of DCs and include pathogen-associated molecular patterns (PAMPs) and damage-associated molecular patterns (DAMPs) (31). This activation enables DCs to present a tumor antigen peptide-MHC class I complex to T cells when contracting with them. DCs could also express secondary signaling necessary for T-cell activation, such as B7 (32). Tumor cells could impair the function of DCs by trapping “danger signals”. For example, stanniocalcin 1 (STC1) could trap DAMPs, leading to the failure of DC activation and T-cell activation. This eventually contributes to tumor immune escape (33). Furthermore, BATF3 DCs are the primary source of CXC-chemokine ligand 9 and 10 (CXCL9 and CXCL10), two key chemokines required for the recruitment of CD8+ T cells to tumors. In the absence of BATF3 DCs, CD8+ T cells would not migrate to the tumor region (34). However, a number of tumor types, including prostate cancer, did not express CXCL9 and CXCL10, which prevented CD8+ effector T cells from activating (35).

Differentiation and recruitment of DCs require various factors like Fms-like tyrosine kinase 3 ligand (FLT3L) and granulocyte-macrophage colony-stimulating factor (GM-CSF) (36). Deficiency of these factors could result in a reduced number of DCs in secondary lymphoid organs and attenuated T-cell immune responses (37). To sum up, impaired DC activation, a lack of DCs, and the overexpression of co-suppressive signals could all lead to T-cell activation failure as DC-T cell crosstalk plays a vital role in naïve T-cell priming.

## T-cell homing dysfunction

Recent evidence has shown that the activation of tumor cell oncogenic pathways is associated with the immune-suppressive TME of “cold tumor”, which causes the potential for ICI therapy resistance. When the WNT pathway was activated, CCL4 expression is downregulated, resulting in decreased recruitment of BATF3 DCs to the TME (38). Also, the WNT pathway activation would cause decreased expression of CXCL9 and CXCL10, making CTLs not being able to recruit to the tumor region. Furthermore, Spranger et al. have found a negative correlation between CD8A expression and activation of the WNT signaling pathway *via* human metastatic carcinoma sample analysis (25). Direct injection of BATF3 DCs into the tumor region restored CXCL9 and CXCL10 expression and T-cell infiltration in WNT signaling pathway-positive tumors (39). Activation of the PI3K/AKT pathway could decrease autophagic activity by reducing the lipidation of the autophagosome protein LC3, which inhibits T-cell homing to the cancer site (40). A TCGA dataset analysis also revealed that the expression of T-cell effector molecules (like IFN-γ, granzyme B) was negatively correlated with the activation of the PI3K/AKT pathway in various carcinomas (40). Oncogenic K-RAS mutations might also contribute to tumor-promoting inflammation through the production of inhibitory cytokines (IL-6, IL-8, etc.), the activation of NLRP3 inflammasome, and the release of chemokines (CCL5, CCL9, etc.) (41). In addition, oncogenic MYC signaling could overexpress CD47 and PD-L1 on tumor cells. As a immune checkpoint, CD47 can attach to signal regulatory proteins α(SIRPα) on the surface of macrophages in the tumor microenvironment and deliver “don’t eat me” messages in order to evade immune surveillance (42, 43). Consequently, CD47 becomes an attractive target for immunotherapy. PD-L1 and CD47 are both highly expressed in tumor cells and can be simultaneously regulated by MYC. A high expression of MYC in tumor cells can regulate the tumor microenvironment by acting on innate immune cells and adaptive immune cells as well as cytokines, and activation of MYC can upregulate the expression of CD47 and PD-L1 leading to immunosuppression and tumor proliferation (MYC can directly act on the promoters of CD47 and PD-L1 and then regulate their mRNA and protein expression levels) (44).

The immunosuppressive cells at tumor sites could also prevent T-cell homing in “cold tumors”. For example, CXCL12, generated by cancer-associated fibroblasts (CAF), could inhibit T-cell infiltration in the tumor region (45). Moreover, CTLs could not reach the edge of “cold” tumors because they could be trapped within the stroma of the tumor or in the peri-tumoral tissue because of the unique immunosuppressive structure built by immunosuppressive cells, like tumor-associated macrophages (TAM), cancer-associated fibroblasts (CAF), regulatory T cells (Tregs), and myeloid-derived suppressor cells (MDSCs) (45) (**Supplementary Figure S1**).

## Immune landscape of prostate cancer

Prostate cancer is considered to be a “cold” tumor as the tumor mutation burden is low, causing a lower number of T-cell infiltrations (46). Prostate cancer also presents a lot of hypoxic zones, which seriously affect the antitumor function of T cells by a variety of mechanisms including the depletion of essential nutrients, abnormal angiogenesis, increased expression of adenosine, acidic pH, immunosuppressive transforming growth factor β (TGF β), and upregulation of PD-L1 (47, 48). Moreover, hypoxic zones could attract immature myeloid cells into the tumor region and turn into myeloid-derived suppressor cells (MDSCs) and tumor-associated macrophages, making the TME of prostate cancer even more immunosuppressed (47). In fact, the immune cells that are part of the prostate tumor microenvironment are frequently characterized by an anergic and immunosuppressive phenotype and include regulatory T cells (Tregs), M2-polarized tumor-associated macrophages (TAMs), and myeloid-derived suppressor cells (MDSCs) (49).

Furthermore, the expression of major histocompatibility complex (MHC) class I, a molecule presenting antigenic protein fragments to cytotoxic T cells, is lost or diminished in prostate cancer (50, 51). Also, PTEN, whose existence could improve the immunosuppressive tumor microenvironment, is frequently downregulated (52), leading to a decrease in the response to immunotherapy in prostate cancer. Single-cell analysis revealed that CD4+ FOXP3+ CD25+ T cells and CD8+ FOXP3+ CD25+ T cells are the main T-cell types in prostate cancer. However, FOXP3+ T cells are immunosuppressive regulatory T cells that both inhibit naive T-cell proliferation and produce inhibitory cytokines, like IL-6 and IL-8 (53, 54).

## Radiotherapy in prostate cancer

Radiotherapy is considered to be one of the most effective therapies for prostate cancer. There are mainly two techniques applied to deliver radiations to the prostate cancer area, namely, brachytherapy (the use of seeds placed in the body) and external beam (projecting energy through the skin) (55).

Brachytherapy means the direct placement of radioactive sources into the prostate gland under the guidance of transrectal ultrasound. There are two dose rates, low dose rate and high dose rate. The low dose rate refers to the permanent implantation of seeds in the prostate tissue so that the seeds project radioactivity gradually. This dose is mainly used in clinics (56). The high dose rate refers to a radiation therapy whose dosage is high enough to possibly lead to leakage to surrounding organs from the prostate tissues. As a result, the high dose rates are rarely applied in the clinic. The advantage of brachytherapy is that the whole process is convenient for patients as it only takes a day or less and no further intervention is needed (57). In addition, the erectile function would not be affected due to brachytherapy (58).

External beam radiation therapy (EBRT) is a commonly used local treatment of tumors in which strong X-ray would emit specifically targeting the prostate tissues (59). Its radiation dose is high (higher than brachytherapy), whereas its radiation would emit less to the surrounding tissues. Radiotherapy could be used in not only patients with localized prostate cancer but also patients with bone metastasis or with metastatic castration-resistant disease (60).

Combination therapy of radiation therapy and androgen deprivation therapy (ADT) is considered to be effective for patients with prostate cancer (61). It is reported that this combination has more advantages than surgical therapy. There are lower risks, such as hemorrhage, myocardial infarction, pulmonary embolism, urine incontinence, and erectile dysfunction, when treating prostate cancer in its early stages (62). This evidence also proved the concept that radiation therapy could be used in company with other therapy in patients with prostate cancer.

## Radiotherapy could “heat” prostate cancer

The conventional goal of radiation in the treatment of cancer is to use high-energy particles to cause deadly DNA damage in tumor cells, causing the cancer cells to eventually perish (63). Recently, the ability of radiotherapy to cause an antitumor immune response has drawn large attention as cancer immune therapy has achieved great success in clinics (64). Studies have proved that radiotherapy could cause immunogenic cell death and cellular stress, which could increase the exposure of tumor-associated antigens and damage-associated molecular patterns (DAMPs) (65). Elevated tumor-associated antigens and DAMPs could activate DCs. Furthermore, DCs could present tumor-associated antigens to CD8+ T cells and help recruit CD8+ T cells into the TME to enhance the antitumor responses (66).

Furthermore, the interferon genes (STING) pathway would be activated in the presence of DAMPs. Production of interferon gene pathways, including type I interferons (IFN-α and IFN-β), could further activate antitumor immune responses (67). Both preclinical and clinical studies validated that radiotherapy could increase antitumor immune cells such as CD4+, CD8+ T, cytotoxic NK, and CD8+CD56+ natural killer T (NKT) cells infiltrating into the TME (68, 69). All of these data imply that radiotherapy may transform “cold” cancers with low immunogenicity and immune cell infiltration into “hot” tumors with dense immune cell infiltration.

Meanwhile, elevated interferon activity *via* radiotherapy could upregulate exhaustion molecules such as PD-1 and PD-L1 (70, 71). Therefore, the combination of radiotherapy plus immune checkpoint inhibitor would be necessary to block the elevated exhaustion molecules, thus avoiding cancer immune escape and enhancing the therapeutic effect of radiotherapy (**Supplementary Figure S2**).

## Clinical studies exploring radiotherapy plus immune checkpoint blockade

There are several clinical trials exploring the therapeutic effect of radiotherapy plus ICI in treating cancers.

A phase 1 study combined anti-PD-1/PD-L1 therapy with stereotactic body radiotherapy (SBRT); finding the combination could be well-tolerable by patients (72). There are five phase 1/2 studies that also showed that anti-PD-1/PD-L1 therapy plus radiotherapy was tolerable for patients with advanced non-small cell lung cancer (NSCLC), head and neck squamous cell carcinoma (HNSCC), and small cell lung cancer (SCLC) (73–77). These findings indicate that the combination of radiotherapy plus ICI is feasible for cancer treatment.

As this field is in its preliminary stages, most studies have shown encouraging efficiencies in patients treated with combination of radiotherapy and ICI. Most combinations of radiotherapy and ICI are based on stereotactic body radiotherapy (SBRT), which could precisely deliver ablative doses of radiation in image-guided and intensity-modulated manners (78). A phase 2 study in resectable non-small-cell lung cancer showed that patients who received 24-Gy SBRT before durvalumab (PD-1 antibody) treatment demonstrated a higher objective response rate (ORR) (53.5%) than patients who only received durvalumab or SBRT (6.7%) (79); however, long-term prognoses (such as OS, PFS) were not recorded in the study. A phase 3 study found that stage III NSCLC patients undergoing radiotherapy and durvalumab acquired a markedly improved progression-free survival (PFS) (17.2 *vs*. 5.6 months) and overall survival (OS) (The 12-month overall survival rate was 83.1% in the durvalumab group, as compared with 75.3% in the placebo group. The 24-month overall survival rate was 66.3% in the durvalumab group, as compared with 55.6% in the placebo group.) compared with those who only received radiotherapy (80). Tai et al. showed that combination of Y-90-RE with nivolumab had an optimistic ORR of 31% in patients with advanced hepatocellular carcinoma (HCC) (81). Chiang et al. reported an astonishing 100% ORR in five patients treated with SBRT followed by nivolumab for large unresectable HCC; another case report showed complete pathological response following Y-90-RE and nivolumab bridging therapy prior to partial hepatectomy (82, 83). Another phase I trial that evaluated liver/lung SBRT with ipilimumab reported that 23% of patients experienced clinical benefit which corresponded to an increase in CD8+ T cells and CD8+/CD4+ ratios (84). Although these findings are promising, these trials only recruited limited patients in only one medical center.

There are also trials that have been carried out to investigate the interaction of radiation and ICI therapy in prostate cancer. Slovin et al. explored the combination of ipilimumab (anti-CTLA-4) and radiotherapy in patients with metastatic castration-resistant prostate cancer, finding that ipilimumab 10 mg/kg plus radiotherapy suggested clinical antitumor activity with disease control and manageable adverse events (85); in addition, the trial suggested that treatment with ipilimumab improved progression-free survival compared with placebo (median 4.0 *vs*. 3.1 months in the placebo group; p < 0.0001). Two phase 3 studies have evaluated the therapeutic effect of ipilimumab plus radiotherapy in patients with metastatic castration-resistant prostate cancer (86, 87). In a preplanned long-term analysis including 799 patients, ipilimumab plus radiotherapy improved the overall survival of these patients significantly. The OS rates at 3, 4, and 5 years were approximately two to three times higher than that of patients receiving only radiotherapy (87).

PD-L1 expression was observed in serial biopsy samples in a phase 1 trial of nivolumab in combination with external beam radiation therapy and brachytherapy in patients with grade 5 prostate cancer and increased immune infiltration. A strong immune infiltrate was observed in the tissues, with an increase in CD8+ and FOXP3+/CD4+ T cells in the tissues and CD4+ effector T cells in the peripheral blood (88). Although a potentially T-cell-mediated antitumor effect of ipilimumab was observed in 13%–23% of men enrolled in the two trials, these data suggest that only a minority of men with mCRPC derive a benefit from the single-agent ipilimumab. Based on preclinical data showing that radiotherapy could improve the tumor microenvironment and the antitumor effect of CTLA-4, these studies used radiotherapy as a boost for ipilimumab. The long-term analysis shows that OS is improved with ipilimumab plus RT versus placebo plus RT in patients with post-docetaxel mCRPC, and treatment was associated with a fraction of patients with long-term survival. Although these findings might suggest that the combination of radiotherapy plus ICI would bring benefits to patients with advanced-stage prostate cancer, no definitive phase 3 studies are testing this combination of ICI plus radiotherapy in prostate cancer; more clinical trials should be conducted to confirm its therapeutic effect and other details such as dose, volume, fractionation, and sequence. The current research studies are listed in **Table 1**, which can further prove the efficacy of radiotherapy combined with ICI in prostate cancer. Those studies might also point out patients with which a specific prostate cancer stage could benefit most from using this combination. Furthermore, these studies might also show the association between radiotherapy plus ICI and conventional therapy such as androgen deprivation therapy, answering questions such as whether this strategy is better. Whether this strategy could be used on patients undergoing androgen deprivation therapy and so on.

**Table 1:** Trials of radiotherapy with ICI in prostate cancer.

Trials	Treatment plan			Status	Main outcome	Reference
	Experimental group A	Experimental group B	Experimental group C			
Karim F et al	Radiotherapy + ipilimumab	Ipilimumab	–	Finished	Radiotherapy with ICB improved OS in patients with post-docetaxel mCRPC	(84)
	SRT + pembrolizumab	Pembrolizumab	–	Recruiting	Not published	NCT04931979
	SRT + ipilimumab/nivolumab	Ipilimumab/nivolumab	–	Recruiting	Not published	NCT05655715
	Nivolumab+ brachytherapy + external beam radiation therapy+ ADT	–	–	Recruiting	Nivolumab with brachytherapy is associated with evidence of increased immune infiltration and antitumor activity	NCT03543189
	SRT + REGN2810	SRT + ipilimumab	SRT + REGN2810+ ipilimumab	Recruiting	Not published	NCT03477864
	SRT + nivolumab CDX-301 + poly-ICLC	Nivolumab + CDX-301 + INO-5151	–	Finished	Not published	NCT03835533
	Pembrolizumab + SRT + ADT	–	–	Recruiting	Not published	NCT04569461

ICI, immune checkpoint inhibitor; OS, overall survival; SRT, stereotactic body radiation therapy; ADT, androgen deprivation therapy; REGN2810, anti-PD-1 monoclonal antibody; CDX-301, the dendritic cell growth factor fms-like tyrosine kinase 3 (FLT3) ligand, a dendritic cell mobilizer; Poly-ICLC, a TLR3 agonist; INO-5151, a combination of DNA plasmids encoding interleukin-12 (INO-9012 formulation) and prostate-specific antigen (PSA) and prostate-specific membrane antigen (PSMA) (INO-5150 formulation) to activate a cytotoxic T-lymphocyte for an antitumor response.

## Perspective

To sum up, radiotherapy combined with immune checkpoint blockade may be a more effective combination to enhance the efficacy of ICI monotherapy in the treatment of prostate cancers, since radiotherapy may boost tumor antigen presentation and CTL infiltration into the cancer region. However, there are currently only a few clinical trials focused on the combination of radiotherapy and immune checkpoint blockade for the treatment of prostate cancer. Further clinical trials are expected in the future to validate its efficiency in treating prostate cancer.

## Author contributions

TL, XQ, XL, BX, and JXL conceptualized, wrote, and reviewed the manuscript. JYL, JY, FX, JL, and GY were involved in writing the article.

## References

[1] Bagchi S Yuan R Engleman EG. Immune checkpoint inhibitors for the treatment of cancer: clinical impact and mechanisms of response and resistance. Annu Rev Pathol (2021) 16:223–49. doi: 10.1146/annurev-pathol-042020-042741 33197221

[2] Carlino MS Larkin J Long GV. Immune checkpoint inhibitors in melanoma. Lancet (2021) 398(10304):1002–14. doi: 10.1016/S0140-6736(21)01206-X 34509219

[3] Bonaventura P Shekarian T Alcazer V Valladeau-Guilemond J Valsesia-Wittmann S Amigorena S. Cold tumors: A therapeutic challenge for immunotherapy. Front Immunol (2019) 10:168. doi: 10.3389/fimmu.2019.00168 30800125 PMC6376112

[4] Antonarakis ES Piulats JM Gross-Goupil M Goh J Ojamaa K Hoimes CJ. Pembrolizumab for treatment-refractory metastatic castration-resistant prostate cancer: multicohort, open-label phase II KEYNOTE-199 study. J Clin Oncol (2020) 38(5):395–405. doi: 10.1200/JCO.19.01638 31774688 PMC7186583

[5] Bockorny B Macarulla T Semenisty V Borazanci E Feliu J Ponz-Sarvise M. Motixafortide and pembrolizumab combined to nanoliposomal irinotecan, fluorouracil, and folinic acid in metastatic pancreatic cancer: the COMBAT/KEYNOTE-202 trial. Clin Cancer Res (2021) 27(18):5020–7. doi: 10.1158/1078-0432.CCR-21-0929 34253578

[6] Padron LJ Maurer DM O'Hara MH O'Reilly EM Wolff RA Wainberg ZA. Sotigalimab and/or nivolumab with chemotherapy in first-line metastatic pancreatic cancer: clinical and immunologic analyses from the randomized phase 2 PRINCE trial. Nat Med (2022) 28(6):1167–77. doi: 10.1038/s41591-022-01829-9 PMC9205784 35662283

[7] de Almeida DVP Fong L Rettig MB Autio KA. Immune checkpoint blockade for prostate cancer: niche role or next breakthrough? Am Soc Clin Oncol Educ Book (2020) 40:1–18. doi: 10.1200/EDBK_278853 32343604

[8] Abida W Cheng ML Armenia J Middha S Autio KA Vargas HA. Analysis of the prevalence of microsatellite instability in prostate cancer and response to immune checkpoint blockade. JAMA Oncol (2019) 5(4):471–8. doi: 10.1001/jamaoncol.2018.5801 PMC6459218 30589920

[9] Marcus L Lemery SJ Keegan P Pazdur R. FDA approval summary: pembrolizumab for the treatment of microsatellite instability-high solid tumors. Clin Cancer Res (2019) 25(13):3753–8. doi: 10.1158/1078-0432.CCR-18-4070 30787022

[10] Zhu S Zhang T Zheng L Liu H Song W Liu D. Combination strategies to maximize the benefits of cancer immunotherapy. J Hematol Oncol (2021) 14(1):156. doi: 10.1186/s13045-021-01164-5 34579759 PMC8475356

[11] Liu YT Sun ZJ. Turning cold tumors into hot tumors by improving T-cell infiltration. Theranostics (2021) 11(11):5365–86. doi: 10.7150/thno.58390 PMC8039952 33859752

[12] Zhang J Huang D Saw PE Song E. Turning cold tumors hot: from molecular mechanisms to clinical applications. Trends Immunol (2022) 43(7):523–45. doi: 10.1016/j.it.2022.04.010 35624021

[13] Mortezaee K Najafi M. Immune system in cancer radiotherapy: Resistance mechanisms and therapy perspectives. Crit Rev Oncol Hematol (2021) 157:103180. doi: 10.1016/j.critrevonc.2020.103180 33264717

[14] Mortezaee K. Enriched cancer stem cells, dense stroma, and cold immunity: Interrelated events in pancreatic cancer. J Biochem Mol Toxicol (2021) 35(4):e22708. doi: 10.1002/jbt.22708 33491255

[15] Mishra R Haldar S Placencio V Madhav A Rohena-Rivera K Agarwal P. Stromal epigenetic alterations drive metabolic and neuroendocrine prostate cancer reprogramming. J Clin Invest. (2018) 128(10):4472–84. doi: 10.1172/JCI99397 PMC6159981 30047926

[16] Jiao S Subudhi SK Aparicio A Ge Z Guan B Miura Y. Differences in tumor microenvironment dictate T helper lineage polarization and response to immune checkpoint therapy. Cell (2019) 179(5):1177–1190 e13. doi: 10.1016/j.cell.2019.10.029 31730856

[17] Maughan BL Pal SK Gill D Boucher K Martin C Salgia M. Modulation of premetastatic niche by the vascular endothelial growth factor receptor tyrosine kinase inhibitor pazopanib in localized high-risk prostate cancer followed by radical prostatectomy: A phase II randomized trial. Oncologist (2018) 23(12):1413–e151. doi: 10.1634/theoncologist.2018-0652 30575560 PMC6292560

[18] Wu Z Chen H Luo W Zhang H Li G Zeng F. The landscape of immune cells infiltrating in prostate cancer. Front Oncol (2020) 10:517637. doi: 10.3389/fonc.2020.517637 33194581 PMC7658630

[19] Redman JM Steinberg SM Gulley JL. Quick efficacy seeking trial (QuEST1): a novel combination immunotherapy study designed for rapid clinical signal assessment metastatic castration-resistant prostate cancer. J Immunother Cancer. (2018) 6(1):91. doi: 10.1186/s40425-018-0409-8 30227893 PMC6145343

[20] McLaughlin M Patin EC Pedersen M Wilkins A Dillon MT Melcher AA. Inflammatory microenvironment remodelling by tumour cells after radiotherapy. Nat Rev Cancer. (2020) 20(4):203–17. doi: 10.1038/s41568-020-0246-1 32161398

[21] Alsaab HO Sau S Alzhrani R Tatiparti K Bhise K Kashaw SK. PD-1 and PD-L1 checkpoint signaling inhibition for cancer immunotherapy: mechanism, combinations, and clinical outcome. Front Pharmacol (2017) 8:561. doi: 10.3389/fphar.2017.00561 28878676 PMC5572324

[22] Havel JJ Chowell D Chan TA. The evolving landscape of biomarkers for checkpoint inhibitor immunotherapy. Nat Rev Cancer. (2019) 19(3):133–50. doi: 10.1038/s41568-019-0116-x PMC6705396 30755690

[23] Chen DS Mellman I. Oncology meets immunology: the cancer-immunity cycle. Immunity (2013) 39(1):1–10. doi: 10.1016/j.immuni.2013.07.012 23890059

[24] Mouw KW Goldberg MS Konstantinopoulos PA D'Andrea AD. DNA damage and repair biomarkers of immunotherapy response. Cancer Discovery (2017) 7(7):675–93. doi: 10.1158/2159-8290.CD-17-0226 PMC5659200 28630051

[25] McGrail DJ Federico L Li Y Dai H Lu Y Mills GB. Multi-omics analysis reveals neoantigen-independent immune cell infiltration in copy-number driven cancers. Nat Commun (2018) 9(1):1317. doi: 10.1038/s41467-018-03730-x 29615613 PMC5882811

[26] Valero C Lee M Hoen D Zehir A Berger MF Seshan VE. Response rates to anti-PD-1 immunotherapy in microsatellite-stable solid tumors with 10 or more mutations per megabase. JAMA Oncol (2021) 7(5):739–43. doi: 10.1001/jamaoncol.2020.7684 PMC7893543 33599686

[27] McGrail DJ Pilie PG Rashid NU Voorwerk L Slagter M Kok M. High tumor mutation burden fails to predict immune checkpoint blockade response across all cancer types. Ann Oncol (2021) 32(5):661–72. doi: 10.1016/j.annonc.2021.02.006 PMC8053682 33736924

[28] Seliger B. Molecular mechanisms of MHC class I abnormalities and APM components in human tumors. Cancer Immunol Immunother. (2008) 57(11):1719–26. doi: 10.1007/s00262-008-0515-4 PMC11030176 18408926

[29] Sharma P Hu-Lieskovan S Wargo JA Ribas A. Primary, adaptive, and acquired resistance to cancer immunotherapy. Cell (2017) 168(4):707–23. doi: 10.1016/j.cell.2017.01.017 PMC5391692 28187290

[30] Torrejon DY Abril-Rodriguez G Champhekar AS Tsoi J Campbell KM Kalbasi A. Overcoming genetically based resistance mechanisms to PD-1 blockade. Cancer Discovery (2020) 10(8):1140–57. doi: 10.1158/2159-8290.CD-19-1409 PMC7416458 32467343

[31] Takeuchi O Akira S. Pattern recognition receptors and inflammation. Cell (2010) 140(6):805–20. doi: 10.1016/j.cell.2010.01.022 20303872

[32] Logue EC Sha WC. CD28-B7 bidirectional signaling: a two-way street to activation. Nat Immunol (2004) 5(11):1103–5. doi: 10.1038/ni1104-1103 15496947

[33] Lin H Kryczek I Li S Green MD Ali A Hamasha R. Stanniocalcin 1 is a phagocytosis checkpoint driving tumor immune resistance. Cancer Cell (2021) 39(4):480–493 e6. doi: 10.1016/j.ccell.2020.12.023 33513345 PMC8044011

[34] Spranger S Dai D Horton B Gajewski TF. Tumor-residing batf3 dendritic cells are required for effector T cell trafficking and adoptive T cell therapy. Cancer Cell (2017) 31(5):711–723 e4. doi: 10.1016/j.ccell.2017.04.003 28486109 PMC5650691

[35] Tokunaga R Zhang W Naseem M Puccini A Berger MD Soni S. CXCL9, CXCL10, CXCL11/CXCR3 axis for immune activation - A target for novel cancer therapy. Cancer Treat Rev (2018) 63:40–7. doi: 10.1016/j.ctrv.2017.11.007 PMC5801162 29207310

[36] Demaria O Cornen S Daeron M Morel Y Medzhitov R Vivier E. Harnessing innate immunity in cancer therapy. Nature (2019) 574(7776):45–56. doi: 10.1038/s41586-019-1593-5 31578484

[37] Kingston D Schmid MA Onai N Obata-Onai A Baumjohann D Manz MG. The concerted action of GM-CSF and Flt3-ligand on in *vivo* dendritic cell homeostasis. Blood (2009) 114(4):835–43. doi: 10.1182/blood-2009-02-206318 19465690

[38] Spranger S Bao R Gajewski TF. Melanoma-intrinsic beta-catenin signalling prevents anti-tumour immunity. Nature (2015) 523(7559):231–5. doi: 10.1038/nature14404 25970248

[39] Gajos-Michniewicz A Czyz M. WNT signaling in melanoma. Int J Mol Sci (2020) 21(14):4852. doi: 10.3390/ijms21144852 32659938 PMC7402324

[40] Peng W Chen JQ Liu C Malu S Creasy C Tetzlaff MT. Loss of PTEN promotes resistance to T cell-mediated immunotherapy. Cancer Discovery (2016) 6(2):202–16. doi: 10.1158/2159-8290.CD-15-0283 PMC4744499 26645196

[41] Hamarsheh S Gross O Brummer T Zeiser R. Immune modulatory effects of oncogenic KRAS in cancer. Nat Commun (2020) 11(1):5439. doi: 10.1038/s41467-020-19288-6 33116132 PMC7595113

[42] Jaiswal S Jamieson CH Pang WW Park CY Chao MP Majeti R. CD47 is upregulated on circulating hematopoietic stem cells and leukemia cells to avoid phagocytosis. Cell (2009) 138(2):271–85. doi: 10.1016/j.cell.2009.05.046 PMC2775564 19632178

[43] Majeti R Chao MP Alizadeh AA Pang WW Jaiswal S Gibbs KD Jr.. CD47 is an adverse prognostic factor and therapeutic antibody target on human acute myeloid leukemia stem cells. Cell (2009) 138(2):286–99. doi: 10.1016/j.cell.2009.05.045 PMC2726837 19632179

[44] Casey SC Tong L Li Y Do R Walz S Fitzgerald KN. MYC regulates the antitumor immune response through CD47 and PD-L1. Science (2016) 352(6282):227–31. doi: 10.1126/science.aac9935 PMC4940030 26966191

[45] Bassani-Sternberg M Digklia A Huber F Wagner D Sempoux C Stevenson BJ. A phase Ib study of the combination of personalized autologous dendritic cell vaccine, aspirin, and standard of care adjuvant chemotherapy followed by nivolumab for resected pancreatic adenocarcinoma-A proof of antigen discovery feasibility in three patients. Front Immunol (2019) 10:1832. doi: 10.3389/fimmu.2019.01832 31440238 PMC6694698

[46] Berger MF Lawrence MS Demichelis F Drier Y Cibulskis K Sivachenko AY. The genomic complexity of primary human prostate cancer. Nature (2011) 470(7333):214–20. doi: 10.1038/nature09744 PMC3075885 21307934

[47] Chouaib S Noman MZ Kosmatopoulos K Curran MA. Hypoxic stress: obstacles and opportunities for innovative immunotherapy of cancer. Oncogene (2017) 36(4):439–45. doi: 10.1038/onc.2016.225 PMC5937267 27345407

[48] Jayaprakash P Ai M Liu A Budhani P Bartkowiak T Sheng J. Targeted hypoxia reduction restores T cell infiltration and sensitizes prostate cancer to immunotherapy. J Clin Invest. (2018) 128(11):5137–49. doi: 10.1172/JCI96268 PMC6205399 30188869

[49] Krueger TE Thorek DLJ Meeker AK Isaacs JT Brennen WN. Tumor-infiltrating mesenchymal stem cells: Drivers of the immunosuppressive tumor microenvironment in prostate cancer? Prostate (2019) 79(3):320–30. doi: 10.1002/pros.23738 PMC6549513 30488530

[50] Sanda MG Restifo NP Walsh JC Kawakami Y Nelson WG Pardoll DM. Molecular characterization of defective antigen processing in human prostate cancer. J Natl Cancer Inst (1995) 87(4):280–5. doi: 10.1093/jnci/87.4.280 PMC2104544 7707419

[51] Bander NH Yao D Liu H Chen YT Steiner M Zuccaro W. MHC class I and II expression in prostate carcinoma and modulation by interferon-alpha and -gamma. Prostate (1997) 33(4):233–9. doi: 10.1002/(sici)1097-0045(19971201)33:4<233::aid-pros2>3.0.co;2-i 9397194

[52] Jamaspishvili T Berman DM Ross AE Scher HI De Marzo AM Squire JA. Clinical implications of PTEN loss in prostate cancer. Nat Rev Urol. (2018) 15(4):222–34. doi: 10.1038/nrurol.2018.9 PMC7472658 29460925

[53] Miller AM Lundberg K Ozenci V Banham AH Hellstrom M Egevad L. CD4+CD25high T cells are enriched in the tumor and peripheral blood of prostate cancer patients. J Immunol (2006) 177(10):7398–405. doi: 10.4049/jimmunol.177.10.7398 17082659

[54] Kiniwa Y Miyahara Y Wang HY Peng W Peng G Wheeler TM. CD8+ Foxp3+ regulatory T cells mediate immunosuppression in prostate cancer. Clin Cancer Res (2007) 13(23):6947–58. doi: 10.1158/1078-0432.CCR-07-0842 18056169

[55] Baskar R Lee KA Yeo R Yeoh KW. Cancer and radiation therapy: current advances and future directions. Int J Med Sci (2012) 9(3):193–9. doi: 10.7150/ijms.3635 PMC3298009 22408567

[56] Potosky AL Legler J Albertsen PC Stanford JL Gilliland FD Hamilton AS. Health outcomes after prostatectomy or radiotherapy for prostate cancer: results from the Prostate Cancer Outcomes Study. J Natl Cancer Inst (2000) 92(19):1582–92. doi: 10.1093/jnci/92.19.1582 11018094

[57] Sekhoacha M Riet K Motloung P Gumenku L Adegoke A Mashele S. Prostate cancer review: genetics, diagnosis, treatment options, and alternative approaches. Molecules (2022) 27(17):5730. doi: 10.3390/molecules27175730 36080493 PMC9457814

[58] Stember DS Mulhall JP. The concept of erectile function preservation (penile rehabilitation) in the patient after brachytherapy for prostate cancer. Brachytherapy (2012) 11(2):87–96. doi: 10.1016/j.brachy.2012.01.002 22330103

[59] Breitkreutz DY Weil MD Bazalova-Carter M. External beam radiation therapy with kilovoltage x-rays. Phys Med (2020) 79:103–12. doi: 10.1016/j.ejmp.2020.11.001 33221545

[60] Morris MJ Fong L Petrylak DP Sartor AO Higano CS Pagliaro LC. Safety and clinical activity of atezolizumab (atezo) + radium-223 dichloride (r-223) in 2L metastatic castration-resistant prostate cancer (mCRPC). Results phase Ib Clin trial (2020) 38(15_suppl):5565–5. doi: 10.1200/JCO.2020.38.15_suppl.5565

[61] Spek A Graser A Habl G Muacevic A Fuerweger C Seitz M. Single-fraction image-guided robotic radiosurgery efficiently controls local prostate cancer recurrence after radical prostatectomy. BJUI Compass. (2020) 1(4):139–45. doi: 10.1002/bco2.32 PMC8988633 35474939

[62] Mellman I Coukos G Dranoff G. Cancer immunotherapy comes of age. Nature (2011) 480(7378):480–9. doi: 10.1038/nature10673 PMC3967235 22193102

[63] Arnold KM Flynn NJ Raben A Romak L Yu Y Dicker AP. The impact of radiation on the tumor microenvironment: effect of dose and fractionation schedules. Cancer Growth Metastasis. (2018) 11:1179064418761639. doi: 10.1177/1179064418761639 29551910 PMC5846913

[64] Reynders K Illidge T Siva S Chang JY De Ruysscher D. The abscopal effect of local radiotherapy: using immunotherapy to make a rare event clinically relevant. Cancer Treat Rev (2015) 41(6):503–10. doi: 10.1016/j.ctrv.2015.03.011 PMC4816218 25872878

[65] Apetoh L Ghiringhelli F Tesniere A Obeid M Ortiz C Criollo A. Toll-like receptor 4-dependent contribution of the immune system to anticancer chemotherapy and radiotherapy. Nat Med (2007) 13(9):1050–9. doi: 10.1038/nm1622 17704786

[66] Sauter B Albert ML Francisco L Larsson M Somersan S Bhardwaj N. Consequences of cell death: exposure to necrotic tumor cells, but not primary tissue cells or apoptotic cells, induces the maturation of immunostimulatory dendritic cells. J Exp Med (2000) 191(3):423–34. doi: 10.1084/jem.191.3.423 PMC2195816 10662788

[67] Deng L Liang H Xu M Yang X Burnette B Arina A. STING-dependent cytosolic DNA sensing promotes radiation-induced type I interferon-dependent antitumor immunity in immunogenic tumors. Immunity (2014) 41(5):843–52. doi: 10.1016/j.immuni.2014.10.019 PMC5155593 25517616

[68] Chew V Lee YH Pan L Nasir NJM Lim CJ Chua C. Immune activation underlies a sustained clinical response to Yttrium-90 radioembolisation in hepatocellular carcinoma. Gut (2019) 68(2):335–46. doi: 10.1136/gutjnl-2017-315485 PMC6352403 29440463

[69] Lugade AA Sorensen EW Gerber SA Moran JP Frelinger JG Lord EM. Radiation-induced IFN-gamma production within the tumor microenvironment influences antitumor immunity. J Immunol (2008) 180(5):3132–9. doi: 10.4049/jimmunol.180.5.3132 18292536

[70] Jacquelot N Yamazaki T Roberti MP Duong CPM Andrews MC Verlingue L. Sustained Type I interferon signaling as a mechanism of resistance to PD-1 blockade. Cell Res (2019) 29(10):846–61. doi: 10.1038/s41422-019-0224-x PMC6796942 31481761

[71] Terawaki S Chikuma S Shibayama S Hayashi T Yoshida T Okazaki T. IFN-alpha directly promotes programmed cell death-1 transcription and limits the duration of T cell-mediated immunity. J Immunol (2011) 186(5):2772–9. doi: 10.4049/jimmunol.1003208 21263073

[72] Luke JJ Lemons JM Karrison TG Pitroda SP Melotek JM Zha Y. Safety and clinical activity of pembrolizumab and multisite stereotactic body radiotherapy in patients with advanced solid tumors. J Clin Oncol (2018) 36(16):1611–8. doi: 10.1200/JCO.2017.76.2229 PMC5978468 29437535

[73] Jabbour SK Berman AT Decker RH Lin Y Feigenberg SJ Gettinger SN. Phase 1 trial of pembrolizumab administered concurrently with chemoradiotherapy for locally advanced non-small cell lung cancer: A nonrandomized controlled trial. JAMA Oncol (2020) 6(6):848–55. doi: 10.1001/jamaoncol.2019.6731 PMC7042914 32077891

[74] Powell SF Gold KA Gitau MM Sumey CJ Lohr MM McGraw SC. Safety and efficacy of pembrolizumab with chemoradiotherapy in locally advanced head and neck squamous cell carcinoma: A phase IB study. J Clin Oncol (2020) 38(21):2427–37. doi: 10.1200/JCO.19.03156 PMC7365766 32479189

[75] Peters S Felip E Dafni U Belka C Guckenberger M Irigoyen A. Safety evaluation of nivolumab added concurrently to radiotherapy in a standard first line chemo-radiotherapy regimen in stage III non-small cell lung cancer-The ETOP NICOLAS trial. Lung Cancer. (2019) 133:83–7. doi: 10.1016/j.lungcan.2019.05.001 31200833

[76] Papadopoulos KP Johnson ML Lockhart AC Moore K Falchook GS Formenti SC. First-in-human study of cemiplimab alone or in combination with radiotherapy and/or low-dose cyclophosphamide in patients with advanced malignancies. Clin Cancer Res (2020) 26(5):1025–33. doi: 10.1158/1078-0432.CCR-19-2609 31796520

[77] Welsh JW Heymach JV Guo C Menon H Klein K Cushman TR. Phase 1/2 trial of pembrolizumab and concurrent chemoradiation therapy for limited-stage SCLC. J Thorac Oncol (2020) 15(12):1919–27. doi: 10.1016/j.jtho.2020.08.022 PMC10600713 32916308

[78] Tree AC Khoo VS Eeles RA Ahmed M Dearnaley DP Hawkins MA. Stereotactic body radiotherapy for oligometastases. Lancet Oncol (2013) 14(1):e28–37. doi: 10.1016/S1470-2045(12)70510-7 23276369

[79] Altorki NK McGraw TE Borczuk AC Saxena A Port JL Stiles BM. Neoadjuvant durvalumab with or without stereotactic body radiotherapy in patients with early-stage non-small-cell lung cancer: a single-centre, randomised phase 2 trial. Lancet Oncol (2021) 22(6):824–35. doi: 10.1016/S1470-2045(21)00149-2 34015311

[80] Antonia SJ Villegas A Daniel D Vicente D Murakami S Hui R. Overall survival with durvalumab after chemoradiotherapy in stage III NSCLC. N Engl J Med (2018) 379(24):2342–50. doi: 10.1056/NEJMoa1809697 30280658

[81] Tai WM Loke KSH Gogna A Tan SH Ng DCE Hennedige TP. A phase II open-label, single-center, nonrandomized trial of Y90-radioembolization in combination with nivolumab in Asian patients with advanced hepatocellular carcinoma: CA 209-678. J Clin Oncol (2020) 38 (15_suppl):4590–4590. doi: 10.1200/JCO.2020.38.15_suppl.4590

[82] Chiang CL Chan ACY Chiu KWH Kong FS. Combined stereotactic body radiotherapy and checkpoint inhibition in unresectable hepatocellular carcinoma: A potential synergistic treatment strategy. Front Oncol (2019) 9:1157. doi: 10.3389/fonc.2019.01157 31799176 PMC6874138

[83] Wehrenberg-Klee E Goyal L Dugan M Zhu AX Ganguli S. Y-90 radioembolization combined with a PD-1 inhibitor for advanced hepatocellular carcinoma. Cardiovasc Intervent Radiol (2018) 41(11):1799–802. doi: 10.1007/s00270-018-1993-1 29845347

[84] Tang C Welsh JW de Groot P Massarelli E Chang JY Hess KR. Ipilimumab with stereotactic ablative radiation therapy: phase I results and immunologic correlates from peripheral T cells. Clin Cancer Res (2017) 23(6):1388–96. doi: 10.1158/1078-0432.CCR-16-1432 PMC5355002 27649551

[85] Slovin SF Higano CS Hamid O Tejwani S Harzstark A Alumkal JJ. Ipilimumab alone or in combination with radiotherapy in metastatic castration-resistant prostate cancer: results from an open-label, multicenter phase I/II study. Ann Oncol (2013) 24(7):1813–21. doi: 10.1093/annonc/mdt107 PMC3707423 23535954

[86] Kwon ED Drake CG Scher HI Fizazi K Bossi A van den Eertwegh AJ. Ipilimumab versus placebo after radiotherapy in patients with metastatic castration-resistant prostate cancer that had progressed after docetaxel chemotherapy (CA184-043): a multicentre, randomised, double-blind, phase 3 trial. Lancet Oncol (2014) 15(7):700–12. doi: 10.1016/S1470-2045(14)70189-5 PMC4418935 24831977

[87] Fizazi K Drake CG Beer TM Kwon ED Scher HI Gerritsen WR. Final analysis of the ipilimumab versus placebo following radiotherapy phase III trial in postdocetaxel metastatic castration-resistant prostate cancer identifies an excess of long-term survivors. Eur Urol. (2020) 78(6):822–30. doi: 10.1016/j.eururo.2020.07.032 PMC8428575 32811715

[88] Yuan Z Fernandez D Dhillon J Abraham-Miranda J Awasthi S Kim Y. Proof-of-principle Phase I results of combining nivolumab with brachytherapy and external beam radiation therapy for Grade Group 5 prostate cancer: safety, feasibility, and exploratory analysis. Prostate Cancer Prostatic Dis (2021) 24(1):140–9. doi: 10.1038/s41391-020-0254-y PMC7882397 32651467

