# Supplementary figures and images for: Radiotherapy plus immune checkpoint inhibitor in prostate cancer
Source: Front Oncol. 2023 Jul 21;13:1210673. doi: 10.3389/fonc.2023.1210673 (PMC10403272; doi:10.3389/fonc.2023.1210673)

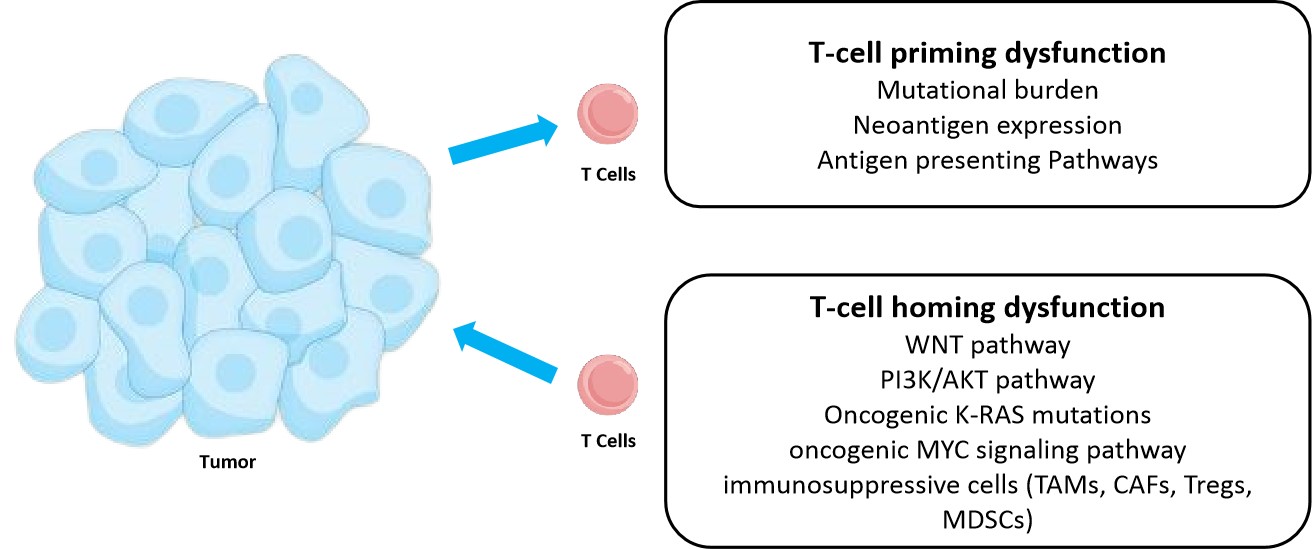

Supplement: Supplementary file 1 [file Image_1.jpg]

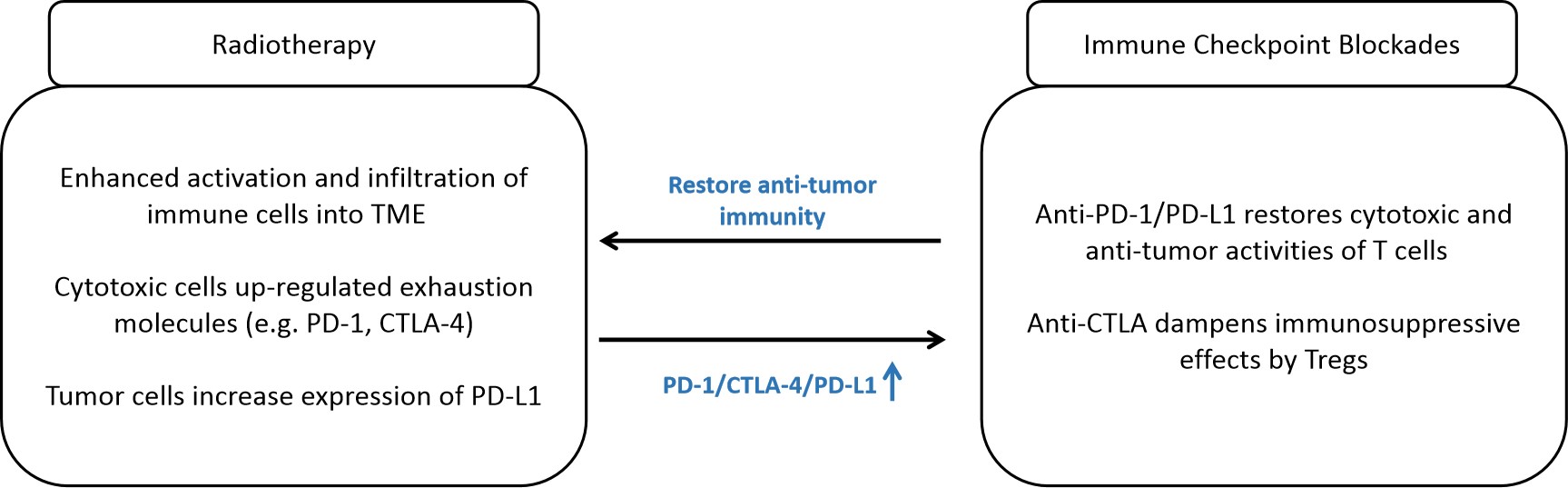

Supplement: Supplementary file 2 [file Image_2.jpg]
